# Evolutionary Modeling of Rate Shifts Reveals Specificity Determinants in HIV-1 Subtypes

**DOI:** 10.1371/journal.pcbi.1000214

**Published:** 2008-11-07

**Authors:** Osnat Penn, Adi Stern, Nimrod D. Rubinstein, Julien Dutheil, Eran Bacharach, Nicolas Galtier, Tal Pupko

**Affiliations:** 1 Department of Cell Research and Immunology, George S. Wise Faculty of Life Sciences, Tel Aviv University, Tel Aviv, Israel; 2 BiRC—Bioinformatics Research Center, University of Aarhus, Århus, Denmark; 3 Institut des Sciences de l'Evolution—CC64, Centre National de la Recherche Scientifique—Université Montpellier 2, Montpelier, France; Utrecht University, Netherlands

## Abstract

A hallmark of the human immunodeficiency virus 1 (HIV-1) is its rapid rate of evolution within and among its various subtypes. Two complementary hypotheses are suggested to explain the sequence variability among HIV-1 subtypes. The first suggests that the functional constraints at each site remain the same across all subtypes, and the differences among subtypes are a direct reflection of random substitutions, which have occurred during the time elapsed since their divergence. The alternative hypothesis suggests that the functional constraints themselves have evolved, and thus sequence differences among subtypes in some sites reflect shifts in function. To determine the contribution of each of these two alternatives to HIV-1 subtype evolution, we have developed a novel Bayesian method for testing and detecting site-specific rate shifts. The RAte Shift EstimatoR (RASER) method determines whether or not site-specific functional shifts characterize the evolution of a protein and, if so, points to the specific sites and lineages in which these shifts have most likely occurred. Applying RASER to a dataset composed of large samples of HIV-1 sequences from different group M subtypes, we reveal rampant evolutionary shifts throughout the HIV-1 proteome. Most of these rate shifts have occurred during the divergence of the major subtypes, establishing that subtype divergence occurred together with functional diversification. We report further evidence for the emergence of a new sub-subtype, characterized by abundant rate-shifting sites. When focusing on the rate-shifting sites detected, we find that many are associated with known function relating to viral life cycle and drug resistance. Finally, we discuss mechanisms of covariation of rate-shifting sites.

## Author Summary

The AIDS epidemic, inflicted by the human immunodeficiency virus (HIV), has already claimed 25 million lives, thus posing a global threat. Since its discovery, several HIV subtypes have emerged, characterized by distinct genomic sequences and variable geographic locations. Here, we investigate the nature of the genetic differences among the subtypes. The neutral theory of evolution suggests that most genetic differences marginally affect the function of the encoded proteins (hence neutral) and thus occur randomly. Alternatively, changes in protein function are reflected by a pattern of nonrandom genetic differences. To address this issue, we developed a computational method, which studies the differences between sequences of different HIV subtypes, and estimates which of the explanations is more likely. Using a large sample of HIV protein sequences, we discovered that part of the variability among the subtypes is not random and possibly reflects different functional constraints imposed on the subtypes during the course of their evolution. An in-depth inspection of these nonrandom changes revealed a correlation with biological traits, such as drug resistance and mechanisms facilitating viral entry into the host cell. Interestingly, nonrandom changes are also characteristic of a viral strain that recently emerged in the former Soviet Union.

## Introduction

Genomic diversity is a key feature of the Human Immunodeficiency Virus type 1 (HIV-1). This high diversity has resulted in the emergence of several distinct groups of the virus, characterized by distinct DNA sequences. HIV-1 is traditionally classified into 3 groups: M (major), O (outlying), and N (new) [1],[2]. The M group accounts for 90% of reported HIV-1 infections, and is further divided into nine subtypes: A, B, C, D, F, G, H, J, and K, each of which is roughly associated with a specific geographical location. Subtype C accounts for nearly half of all new infections, and predominates in eastern and southern Africa, India, and Nepal. Subtypes A, D, G, H, and K have been detected in different regions of Africa. Subtype F is common in central Africa, South America and east Europe, whereas subtype J is exclusive to Central America. Subtype B is predominant in the western world (Europe, the Americas, Japan, and Australia). As such, subtype B is the most widely studied subtype in the laboratory, despite being responsible for only 12% of global infections [3].

Different HIV-1 subtypes display as much as 20–30% variation in their Env nucleotide sequences [4]. On the other hand, the Pol and Gag sequences of different subtypes display less diversity, since they encode the three crucial enzymes (protease, reverse transcriptase (RT), and integrase) and the viral structural proteins, which are less tolerant to changes. Large differences among subtypes also exist in the accessory and regulatory proteins Nef, Vif, Vpr, Vpu, Rev, and Tat. For example, subtype C encodes a truncated Rev protein and an elongated Vpu protein [5], both of which are functional.

To date, inconclusive evidence exists on the general effect of the high genetic diversity of HIV-1 subtypes on protein functionality (reviewed in [1],[3]). Several studies have found positive Darwinian selection to affect only certain clades in the Env [6],[7], protease, and RT proteins [8],[9]. As well, adaptive coevolutionary events were found to explain some of the variability between subtypes [10]. Recently, differential conservation of position 31 in the Tat protein among different subtypes was found to correlate with different functionality of the this protein in subtype C [11]. On the other hand, several studies have found little to no differences among the subtypes' responses to drug administration on a short term basis [12]–[15], which may mean that there are only negligible differences among subtypes in the functionality of protease and RT, the major targets of drug therapy.

In contrast to these specific isolated cases, a widespread study of the differential patterns at all positions of the HIV-1 proteome across its different subtypes has not yet been undertaken. Here, we describe a global study of the differences among group M subtypes, in an attempt to reveal what drives the evolution of the different subtypes, and what are the functional differences among them, if any. Two mechanisms may explain the observed variability among HIV-1 subtypes. The first, in concordance with the neutral theory of molecular evolution [16], suggests that sequence variability across HIV-1 subtypes can be explained solely by random stochastic changes across its phylogeny, with sequences that diverged early (e.g., from different subtypes) showing more variability than sequences that diverged recently (e.g., from the same subtype). In this scenario, the level of selection operating on a specific site is constant along all lineages, and only evolutionary time accounts for the differences observed. The second conjecture suggests that an additional assumption is required to explain the observed sequence variability. According to this view, in some sites the functional constraints have themselves evolved along the lineages. Thus, some of the observed variability among subtypes reflects changes in the function of specific protein sites.

What is the contribution of each mechanism to the observed variability of HIV-1 sequences? If only stochastic changes explain the variability in HIV-1 sequences, then this variability should be distributed evenly throughout the phylogeny. On the other hand, functional changes characterizing specific subtypes will display unique sequence patterns across the subtypes' phylogeny. Such functional changes in a protein are reflected by shifts in its evolutionary rate [17]–[23]. Accordingly, any of the protein sites may change its rate of evolution across the phylogenetic tree, a process previously termed “heterotachy” [24] or “covarion-like” evolution [25]. This is reflected when one subclade of the tree displays one certain pattern (e.g., a low rate of evolution), while the second subclade displays a different pattern (e.g., a high rate of evolution). Sites displaying such a pattern are indicative of either gain of function in a previously unconstrained site, or equivalently loss of function in a previously constrained site, in specific lineages.

A second, similar pattern reflecting rate shifts is when one subclade of the tree is conserved at a certain position with a certain set of amino acids, while the complementary subclade is also conserved at the same position, yet with a different set of amino acids. This type of pattern is usually termed a content shift, but in essence it often reflects a rate shift. Consider the case in which one subclade is conserved for character “A” while the complementary subclade is conserved for character “B”. Clearly, if we ignore the branch connecting the two sublcades, this position evolves with a zero rate. However, in the branch connecting the two subclades, at least one change must have occurred. Thus, especially if this branch is short, this indicates high rate of substitution per unit evolutionary time. Hence, content shift in this case implies rate shift along the branch separating the two subclades. In general, both these types of rate shifts reflect specialization of a site for a certain function. Such a site is hereby termed a specificity determinant.

Several methods exist for the detection of sites which undergo functional shifts. Some of these methods rely on computing the ratio of non-synonymous to synonymous substitutions (Ka/Ks) across different lineages [7],[26],[27]. The aim of these methods is to detect positive Darwinian selection operating on specific sites and lineages. Alternatively, there are methods which contrast evolutionary rates of amino-acid replacements across different lineages (e.g., [17], [18], [23], [28]–[31]). These methods are not limited to detecting positive Darwinian selection but are rather more suitable for detecting general changes in selective constraints. For example, a site which entirely evolves under neutral evolution in one subtree, while in the complementary subtree it has gained a novel function and is now conserved, will most likely go undetected by methods of positive selection. Furthermore, the advantage of methods searching for rate shifts on the amino-acid level is that they are expected to be less sensitive to biases caused by saturation [32] of synonymous substitutions or by selection operating on silent sites [33].

Here, we describe a method for the detection of rate-shifting sites in a protein across all lineages in the phylogeny. This method, hereby termed RASER (RAte Shift EstimatoR) is based on the likelihood framework, combined with empirical Bayesian inference. One of the main novelties of RASER is that as opposed to previous methods, it does not require pre-specification of the lineages in which the suspected rate shifts have occurred. The method is based on an evolutionary model, which incorporates both among-site rate variability and among-site variability of rate shifts, based on the premise that some sites experience more rate shifts than others. Hence, the underlying evolutionary model of RASER allows more than one rate shift to occur in a site along the phylogeny. The model can be used to perform a likelihood ratio test (LRT) to determine whether the data significantly support rate-shifting sites. Furthermore, using a Bayesian framework, RASER can detect sites with a high posterior probability of rate shift. For these sites, it determines the lineage or lineages in which a rate shift has most probably occurred.

RASER was used in order to test whether the observed variability in HIV-1 sequences can be explained by random patterns of evolution alone or by functional considerations, and was applied to the entire HIV-1 proteome. In all of the nine open reading frames (ORFs) of HIV-1, an abundance of sites were inferred to have experienced a shift in their evolutionary rate, suggesting functional specialization occurred in these proteins. The corresponding lineages in which these rate shifts occurred were determined, and were found to highly correlate with the branching patterns of the different subtypes of HIV-1 group M. Furthermore, many of these inferred rate-shifting sites have been previously shown to be functionally important for the viral life cycle and are involved in drug resistance. These results support the hypothesis that some of the variability observed among the different subtypes is a direct result of differing functionality of protein sites. We discuss the importance of the shift in rates in the context of differences in protein functionality of each subtype.

## Results

We developed an evolutionary model and method, RASER, for the detection of sites that have undergone a shift in their evolutionary rate. The heart of the model is based on the previously developed site-specific-rate variation (SSRV) model [25]. The model was used to analyze a total of 182 HIV-1 genome sequences from seven HIV-1 subtypes of group M (A, B, C, D, F, G, and J). For subtypes H and K no reliable genomic sequences were found (see Methods). Each of the nine ORFs of HIV-1 was analyzed separately. Our results clearly show that all of these nine ORFs significantly support rate shifts as compared to a null model, which does not allow for rate shifts (all *P*-values<10^−10^, well below the significance threshold of 0.0056 after Bonferroni correction; Table 1). This suggests that much of the HIV-1 sequence variability is also driven by functional considerations, and cannot be explained merely by stochastic substitutions across the phylogeny under a constant selective regime.

**Table 1:** Maximum log-likelihood (LL) values for the analysis of the nine HIV-1 ORFs under the rate shift and null models.

HIV-1 ORF	Rate Shift Model LL	Null Model LL	2ΔLL	*P*-Value ( 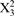 )	Proportion of Rate-Shifting Sites
Env	−94,304.3	−94,782.9	957.2	<10^−20^	0.08
Gag	−28,692.7	−28,867.7	350	<10^−20^	0.06
Nef	−19,049.6	−19,142.1	185	<10^−20^	0.07
Pol	−40,157	−40,364.6	415.2	<10^−20^	0.04
Rev	−10,598.5	−10,712.9	228.8	<10^−20^	0.16
Tat	−9,846.7	−9,936	178.6	<10^−20^	0.15
Vif	−13,406.2	−13,509.7	207	<10^−20^	0.09
Vpr	−6,177.1	−6,208.2	62.2	<10^−10^	0.08
Vpu	−9,511.4	−9,624.3	225.8	<10^−20^	0.30

In order to ascertain the validity of the rate shift model to differentiate between a random pattern of evolution and evolution driven by functional considerations, we conducted simulation studies. To this end, 100 datasets were simulated under the assumption that all the variability in the sequences is due to stochastic substitutions along the phylogeny. By applying LRT, we used RASER to test in how many datasets rate shift was inferred, thus giving an indication of the false positive error rate of the rate shift method on the gene level. We reject the null hypothesis and infer rate shift if the LRT *P*-value is below α = 0.05. Using this cutoff level, the error rate was found to be 3%. At a cutoff level of α = 0.01, the error rate was reduced to zero (note that the maximal *P*-value obtained in the HIV-1 dataset was 10^−10^). We next tested the error rate on the site level, by testing how many sites displayed rate shifts in our simulated data, i.e., how many sites displayed a posterior probability higher than 0.95 in favor of a rate shift (see Methods). Here, we obtained a zero error rate in all of the datasets simulated. All in all, the simulation studies strongly support the notion that the variability across the HIV-1 phylogeny of the different subtypes is functionally driven, and cannot be explained by genetic drift alone.

After establishing that rate shift events are characteristic of all HIV-1 ORFs, we next aimed at identifying the specific sites that contribute to this pattern. Rate-shifting sites were defined as sites displaying a posterior probability higher than 0.95 in favor of a rate shift. A total of 225 rate-shifting sites were detected throughout the HIV-1 proteome (summarized in Table S1). Specifically, Vpu, Rev, and Tat showed an exceptionally high proportion of rate shifts (Table 1). The lowest proportion of rate-shifting sites was observed in Pol and Gag. This is somewhat expected, due to the high level of purifying selection these two genes undergo. Nevertheless, a total of 70 sites displayed significant rate shift in both these genes.

Using the available protein structures of the HIV-1 proteins we explored where rate-shifting sites tend to occur. For the RT protein (Protein Data Bank (PDB) [34] ID 1rtd): 15 of 416 surface sites and only one out of 138 buried sites were found to be rate-shifting (the sites are detailed in Table S1). This difference is statistically significant (*P*-value<0.05; *G*-test), suggesting that the solvent accessible surface of RT is enriched with rate-shifting sites. In all other protein structures, no significant trend was found for the rate-shifting sites.

**Figure 1:**
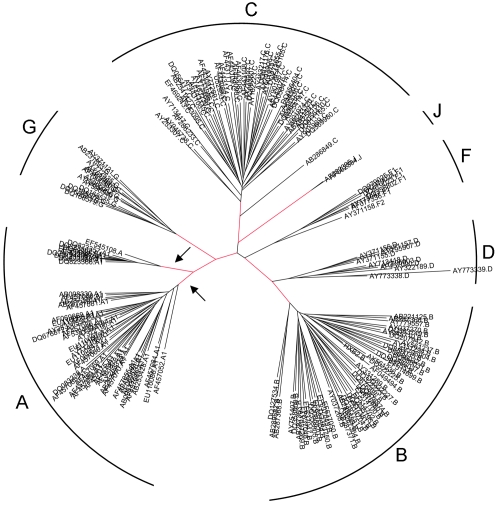
Phylogenetic tree of all nine concatenated datasets of the ORFs. The different subtypes are marked at each subclade of the tree. Branches in red are the top scoring lineages for which rate shifts were found. Arrows mark the two distinct clades of subtype A (see text for details).

We next asked whether this pattern of rate shifts throughout all the nine ORFs can be ascribed to the temporal pattern across the phylogeny, which also represents the divergence into the different subtypes. We thus developed a method based on a Bayesian approach to map significant rate-shifting sites to specific lineages. The method also reports whether a rate shift corresponds to an acceleration or deceleration of the rate at the inferred lineage. Figure 1 shows the top ten lineages for which the most rate-shifting sites were found. The majority of these lineages are ones that separate between different subtypes. Together with the above described results, this result conclusively points to the fact that the sequence-based differences among the subtypes cannot be attributed to random stochastic changes alone, but are, at least in part, a consequence of functional requirements that arose following the emergence of the subtypes. Accordingly, each subtype is characterized by specific specificity determinant sites which display a unique pattern as compared to other subtypes. Table S2 summarizes all the rate-shifting sites for each subtype, according to accelerations and decelerations.

Two lineages with abundant rate shifts are not associated with subtypes, and are within subtype A. According to the phylogeny they clearly distinguish between two divergent groups within this subtype (Figure 1). The larger clade is composed mainly of sequences originating from Africa, and the smaller clade is composed exclusively of sequences originating from the former Soviet Union, where an outbreak of subtype A infections has been reported in injecting drug-users [35]–[38]. We found a total of 25 rate-shifting sites in the lineage leading to this variant, termed IDU-A [39], of which 20 are decelerations. In line with the low genetic diversity reported in IDU-A [38],[39], these sites may be viewed as specificity determinants of this variant, and may represent gain of function of these sites in this variant. All in all, this strengthens the notion that subtype A is in fact composed of two functionally distinct clades, and it may be proposed that this represents an emergence of a novel sub-subtype.

### Correspondence between Functional Sites and Rate-Shifting Sites

To exemplify possible effects of rate shift on the function of HIV-1, we mapped the inferred rate-shifting sites onto an annotation of all functional elements in the HIV-1 genome (available at the Los Alamos HIV sequence database; http://www.hiv.lanl.gov), and performed an additional manual literature search for known functional sites. To the best of our knowledge, there is no database summarizing all literature data on HIV-1 sequence positions, and thus the functional annotation we related to here is non-comprehensive. Nevertheless, 25 rate-shifting sites map to a variety of functional elements at the protein level (summarized in Table 2).

**Table 2:** Rate-shifting sites for which functional annotation is available.

ORF	Encoded Protein	Protein Site^a^	Annotation
Vpr		S77	Mutation implicated in long-term survival [73]
Gag	matrix	E12	Drug resistance associated [74]
	p7 nucleocapsid	G381	Drug resistance associated [74]
		V390	Drug resistance associated [74]
	p6	E460	Binding region of Vpr to p6 [75]
		R490	Binds Vpr, in order to incorporate Vpr into virion nucleocapsid [76]
Pol	protease	E35	Drug resistance associated (e.g., [40],[77])
		M36	Drug resistance associated [42]
Env	gp120	11 sites dispersed in the V2–V4 loops	
		I277	CCR5 binding [78] (Part of V3 loop)
		F287	CCR5 binding [78] (Part of V3 loop)
		V342	CCR5 binding [46]
		R414	CCR5 binding [46]
	gp41	Q32	Luecine/isoleucine Zipper-like sequence, which may be involved in the fusion process to membrane fusion of gp41 [79]
Rev		T34	Part of the RRE binding site [43]
Nef		E62	Acidic region at sites 62–65 (EEEEE)
		E64	

a Protein coordinates are given according to the encoded protein, apart from the p7 and p6 for which the Gag coordinates are given.

**Figure 2:**
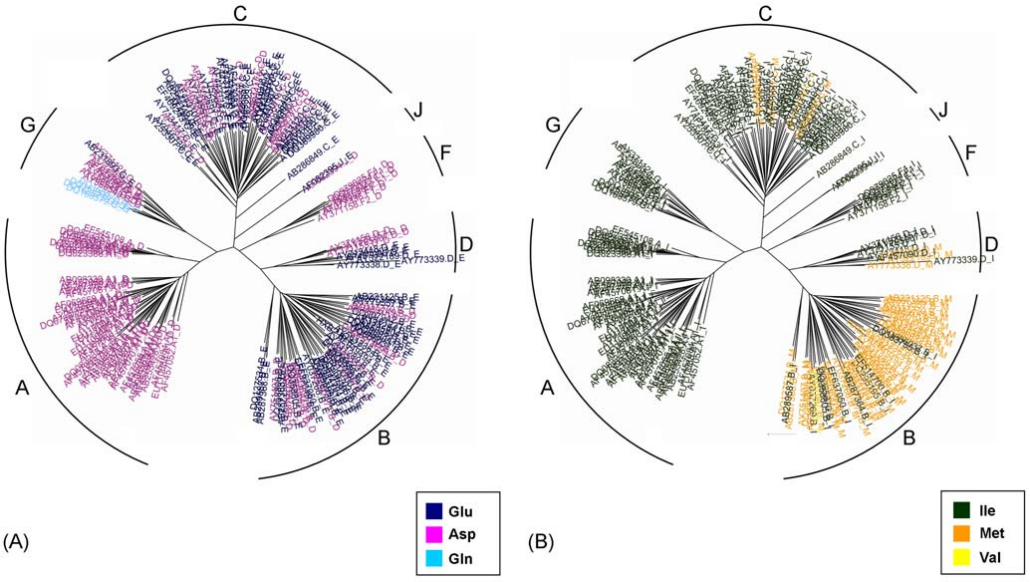
The rate-shifting patterns at sites 35 (A) and 36 (B) of protease, displayed on the phylogenetic tree of all seven subtypes. Each leaf (HIV-1 sequence) is color-coded according to the amino-acid it encodes at this position. Each leaf is labeled by its accession number, subtype (A, B, C, D, F, G, or J), and the encoded residue. The different subtypes are marked at each subclade of the tree. These sites are associated with drug resistance in combination with other sites.

Interestingly, three rate-shifting sites at Gag (sites 12, 381, and 390) and two at Protease (sites 35 and 36) were previously reported to be involved in drug-resistance. These three sites at Gag are non-cleavage sites (i.e., are not cleaved by protease), which contribute to the development of drug resistance against protease inhibitors [38]. These sites display a clear rate shift: sites 12 and 381 are relatively conserved across six of the seven subtypes, and variable in the remaining subtype (for Gag 12 - subtype B is variable, and for Gag 381 - subtype C is variable), while site 390 is conserved across subtypes A and G and variable in the rest of the tree. At site 35 of protease, a mutation from glutamic acid to aspartic acid has been reported as resulting in drug resistance to amprenavir, ritonavir [40], and tipranavir [41] in combination with other sites. Interestingly, aspartic acid completely dominates subtypes A and F, while it is less frequent in all other subtypes (Figure 2A). Similarly, at site 36 of protease, a mutation from methionine to isoleucine contributes to resistance to ritonavir, nelfinavir, and other drug combinations [42]. Once again, isoleucine prevails in almost all subtypes other than subtype B (Figure 2B). Thus, our results suggest caution when administering such drugs since some subtypes may have a predisposition for resistance.

**Figure 3:**
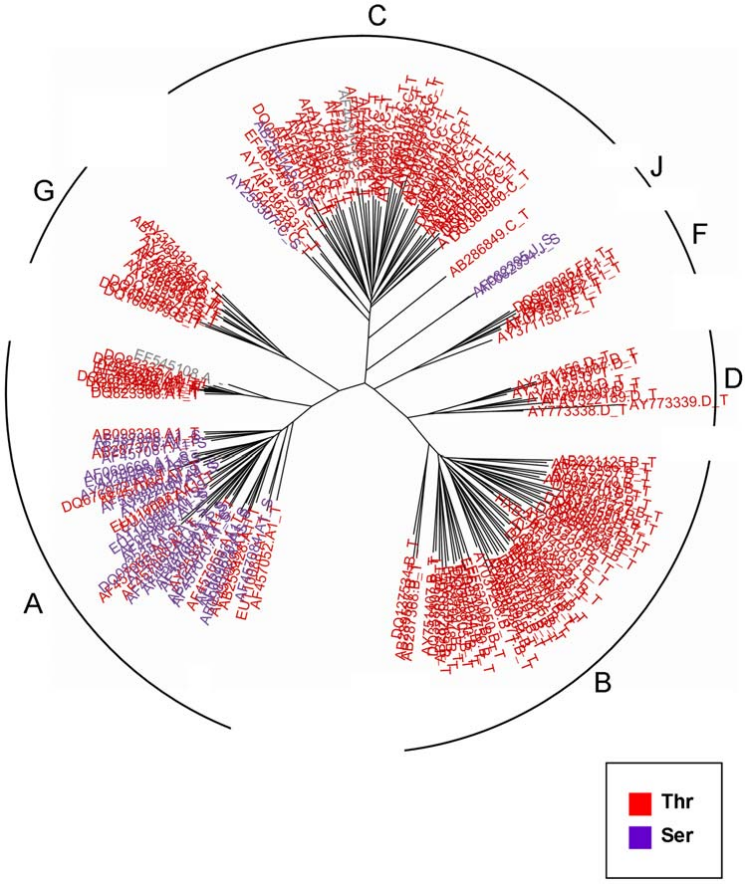
The rate-shifting pattern at site 34 of Rev, displayed on the phylogenetic tree of all seven subtypes. Each leaf (HIV-1 sequence) is color-coded according to the amino-acid it encodes at this position. Each leaf is labeled by its accession number, subtype (A, B, C, D, F, G, or J), and the encoded residue. The different subtypes are marked at each subclade of the tree. This site is part of the RRE binding domain.

Another interesting example of a rate shift at a functional position is site 34 of Rev, which is part of the Rev response element (RRE) binding domain (sites 33–46) [43]. This region in the Rev protein binds the intron-containing viral RNAs, and thus the ribonucleoprotein complex is exported from the nucleus to the cytoplasm. This process is crucial for expression of viral late phase genes that are necessary for viral particle formation [44]. Site 34 in Rev displays a high level of conservation, with threonine encoded at this position throughout the majority of the subtypes (Figure 3). Yet, in subtypes J and the African clade of subtype A, serine is prevalent. Since the two amino acids are quite similar in nature, one might argue that interchanging them has no functional consequence. If so, we would expect both amino-acids to prevail throughout all subtypes. However, it is evident that entire subtypes still “chose” to encode a specific amino-acid at that position. Thus, the shift between the two amino-acids displayed in the above-mentioned clades is likely to represent a genuine functional difference among the subtypes, and in fact may play a role in the binding properties of this region in Rev.

**Figure 4:**
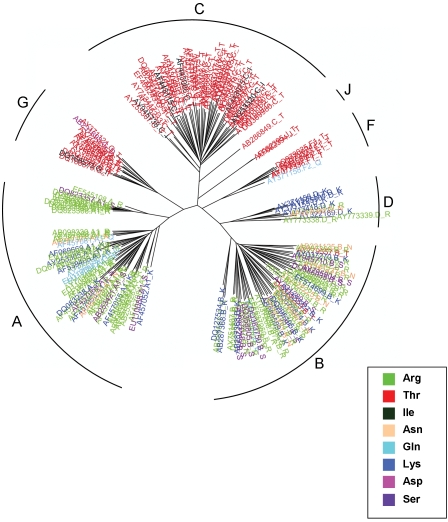
The rate-shifting pattern at site 414 of gp120, displayed on the phylogenetic tree of all seven subtypes. Each leaf (HIV-1 sequence) is color-coded according to the amino-acid it encodes at this position. Each leaf is labeled by its accession number, subtype (A, B, C, D, F, G, or J), and the encoded residue. The different subtypes are marked at each subclade of the tree. This site is involved in CCR5 binding.

Intriguingly, several sites in gp120 that are involved in the co-receptor CCR5 binding were detected as rate-shifting (Table 2), pointing at possible adaptations of different subtypes to different alleles of CCR5. For instance, the CCR5 Δ32 mutation is known to confer reduced susceptibility to the virus in Europe and western Asia [45], and this might affect the pattern of selection pressure acting on these sites. One example of a rate-shifting site affecting CCR5 binding is at position 414 of gp120, which was shown to be involved in CCR5 binding [46]. This site displays several rate shifts across a few of the subtypes (Figure 4), with threonine prevalent at subtypes C, F, J, and G, arginine prevalent at variant IDU-A, and relatively high variability in the rest of the subtypes. Clearly, at this site differing selection constraints operate at each subtype. One may speculate that these subtypes infect patients where a certain allele of CCR5 is more common, and the virus has adapted the gp120 protein to obtain enhanced binding. Future research is required to determine whether rate-shifting positions at subtypes correlate with the populations they infect.

## Discussion

HIV-1 strains were identified more than 20 years ago, and a classification system dividing them into distinct groups and subtypes was formalized in the year 2000 [47]. However, the functional significance of this classification still remains unclear. Since the various subtypes correlate with geographic location, it is natural to postulate that the observed sequence variability is a direct result from stochastic changes among independent lineages (that is, HIV-1 genomes from two different subtypes are variable only because of the long time elapsed since their divergence). Our goal was to test the hypothesis that the sequence divergence also reflects functional divergence. To this end, we developed a methodology for detecting proteins that underwent evolutionary rate shifts, the specificity determinant rate-shifting sites within these proteins, and lineages in which most of these shifts had occurred. Indeed, our method revealed extensive rate shifts among HIV-1 group M subtypes. This strongly suggests that the high variability among the different subtypes is not exclusively a result of stochastic changes, which occurred since the time the subtypes diverged, but also has significant functional consequences.

What is the source of these observed functional shifts? One explanation is that different subtypes are subject to different external (environmental) selective constraints, which are related to their geographical distribution. An example for such an environmental constraint is the selection force exerted by the host adaptive immune system response, coordinated by CTLs and neutralizing antibodies. The CTL response is mediated by human leukocyte antigens (HLAs), which present viral peptides on the surface of infected cells. Different HLA alleles present different peptides, and thus escape-mutations of the virus are expected to correlate with HLA genotypes. Since these genotypes often correspond to different human subpopulations [48],[49], it has been previously suggested that polymorphisms within HIV-1 are associated with HLA genotypes [8], [50]–[52]. However, several of the associations between subtypes and HLA were recently shown to be a result of a founder effect of the subtype [53]. Further complicating this issue is evidence showing there is a fitness cost to the virus due to the escape-mutation at the epitope itself, and that, often, escape will be achieved via a mutation at the region flanking the epitope (affecting antigen processing) (e.g., [54]).

Common to all the explanations for functional requirements is that there is a specific adaptation of the virus to maximize its fitness to its natural human host subpopulation. However, a non adaptive explanation for functional shifts in HIV-1 subtypes can also be suggested: both the function and the fitness of the protein as a whole remain the same, yet different positions in the protein assume different roles in different subtypes, in order to maintain this similar function. If we consider the sequence space as a fitness landscape, there may be two hills with the same or similar fitness. As a simplified example, in the first hill, amino-acid A is fixed in a certain position *i*, allowing the amino acid at position *j* to vary. In the second, equally-fit, hill, amino-acid B is fixed at position *j*, which allows position *i* to vary. In essence, this type of process was originally defined as a “covarion” process [55]. Under this covarion model, the only way to neutrally move from one hill to the other is via A and B at both positions *i* and *j* − a relatively rare event. Most likely, this covarion process will involve several sites which can interchange with a complex terrain of fitness. Thus, dependencies among (two or more) positions might introduce apparent rate shifts that do not change the fitness of the protein, nor do they reflect adaptation at the whole virus level. However, they do reflect functional differences at the single site level, since different sites assume different roles. This explanation is in line with the relative paucity of known functional differences among HIV-1 subtypes, for instance in the context of drug resistance. Nevertheless, lack of evidence for differing functionality on the protein level does not mean such differences do not exist – perhaps not as overwhelming functional differences but as more subtle effects, such as differing inter-molecular interactions. Currently, it is unclear which of the explanations – the “protein adaptation” theory or the “position covarion” theory prevails in the context of HIV-1 subtypes. Most likely, both play an important role in the evolution of these strains.

The rate shift methodology developed here is based on a robust probabilistic framework and can be used to reveal both temporal and spatial evolutionary rate shifts in specific genes, sites, and lineages. One main advantage of the RASER method is that it is statistically based, and the strength of the signal and the sample size are inherently accounted for by taking into account the phylogeny, the number of sequences in each subclade, and the length of the branch separating them. RASER is generic and may be applied to various types of sequence data, ranging from different viral populations, through different phylogenetic taxa, to duplicated genes. For example, an analysis of the rate-shifting sites in avian and human influenza strains could provide valuable information as to the evolution of influenza strains, and therefore their functional adaptations and virulence. As such, RASER can be used to link phenotypic changes with sequence variability. While the vast majority of sequence variability is neutral or slightly deleterious, our method can extract the signal associated with the phenotypic change from the large background stochastic noise.

## Materials and Methods

### An Evolutionary Markov Model for the Detection of Site-Specific Rate Shifts

The most common practice to account for among-site rate variation (ASRV) is to assume that the evolutionary rate *r* at each site is independently sampled from a gamma distribution [56],[57]. A discrete approximation with *k* rate categories is used [58] in order to employ the gamma distribution in the ASRV model. However, this ASRV model assumes that the evolutionary rate is fixed along the phylogeny for a given site. In the SSRV model [25] this assumption is alleviated by allowing the rate at a given site to switch between rate categories rather than being constant. Let *ν* represent the rate at which a site switches between rate categories. This parameter reflects the rate of substitution-rate. The SSRV model is represented as a continuous time Markov process, defined by the instantaneous rate matrix *Q*, where the rate of substitution from state *i* to state *j* (*Q_ij_*) is defined as follows:
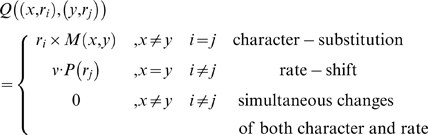
where *M* is any standard rate matrix over any alphabet (nucleotides, amino acids, or codons), *r_i_* and *r_j_* are rates sampled from the discrete gamma distribution, *x* and *y* are alphabet characters, and *P*(*r_j_*) is the prior probability of rate *r_j_*. The diagonal elements of *Q* are determined so that the sum of entries in each row is zero.

In the SSRV model, the υ parameter is assumed to be constant across all sites. Thus, it is implicitly assumed that all sites are potentially rate-shifting sites, and the extent of rate shift is homogenous across all sites. Biological intuition suggests that most sites do not undergo rate shift. However, those few that do, experience this phenomenon at various degrees (i.e., a site may have experienced one or more rate shifts across the phylogeny). Thus, we develop here a model, RASER, in which the rate of rate shifts itself varies among sites, i.e., we assume a distribution over the parameter *ν*. We note that when *ν* = 0, the SSRV model collapses to the ASRV model, and when *ν*→∞ the SSRV model collapses to an equal rates (homogenous) model in which all sites have the same evolutionary rate. In order to account for these two extremes, we use a discretized general gamma distribution (as opposed to the ASRV model, the expectation of the gamma distribution here is not set to 1), with two extra categories to describe *ν* = 0 and *ν*→∞ (in practice, *ν* = 20 was found to approximate homogenous rates well enough, and was used here). The proportions of these two extra categories, *P*(*ν* = 0) and *P*(*ν*→∞), are estimated from the data. Furthermore, to avoid *ν* values which are near these two extremes of 0 and ∞, the gamma distribution is estimated using five fixed categories between 0 and 2 (0.4, 0.8, 1.2, 1.6, 2). We note that using ten fixed categories across the same range yielded essentially the same results and was discarded due to computational considerations. We further use four fixed rate categories (0.25, 0.75, 1.25, 2) to model the among-site rate variation. The use of fixed rate categories was performed in order to avoid the detection of mild rate shifts between similar rate categories, which may occur if rate values are estimated from the data.

### Substitution Matrix

The evolutionary model we develop is general and may incorporate any substitution matrix *M* into it. In this study, the ProtTest software [59] was used to determine the substitution matrix that best fits the data under the Akaike Information Criterion, and this was found to be the HIVb matrix [60].

### Testing for Significant Rate-Shifting Sites

Our methodology for assessing significant rate shifts is similar to the approach for the detection of positive selection at sites in proteins [61]. We first test whether the data significantly support the RASER model using LRT. If so, we report positions supporting rate shift with a posterior probability higher than some cutoff value, here 0.95. As a final stage, we also report the most likely branches at which the rate shift occurred, i.e. the branches with the highest posterior probability of a rate shift occurring there. The details are elaborated in the following sections.

### Likelihood Ratio Test versus a Null Model

To test whether RASER fits the data significantly better than a null model, LRT was performed between the two models. Formally,
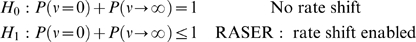
Under RASER, five parameters are assumed (*α* for the gamma rate distribution, *α* and *β* for the gamma distribution over *ν*, *P*(*ν* = 0), and *P*(*ν*→∞)), whereas in the null model only two parameters are assumed (*α* for the rate distribution, and *P*(*ν* = 0)). All parameters are estimated using standard maximized likelihood techniques [62]. Branch lengths are optimized using an expectation-maximization (EM) algorithm. The regularity conditions for the 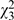 approximation of the LRT are not satisfied, since in essence the parameters *P*(*ν* = 0)+*P*(*ν*→∞) reach a boundary condition. Self and Liang [63] proposed in this case to use a 50∶50 mixture of point mass 0 and *χ* ^2^. However, to avoid errors obtained by small samples, we prefer to be conservative and use 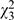 as an approximation.

### Inferring Lineages in Which Rate Shifts Occurred

For sites with a high posterior probability of rate shift, our aim is to detect the lineage or lineages where a rate shift occurred. Thus, we report the three branches which are the most probable candidates at which the rate shift occurred, i.e., those branches with the highest posterior probability of a rate shift. Branches which lead to a leaf in the tree are excluded. To this end, we calculate the posterior probability that a rate shift occurred at each branch. Let us assume a branch which begins with node *A* and ends in node *B*. The posterior probability of a rate shift at this branch will then be:
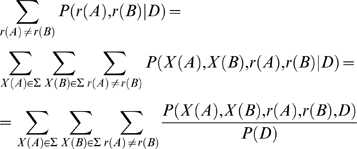
(1)where *r*(*A*) and *r*(*B*) represent the rates at nodes *A* and *B*, respectively, *X*(*A*) and *X*(*B*) represent the character states at these nodes, *Σ* represents the alphabet of the data (in this study, amino acids), and *D* represents the data. The denominator in Equation 1, *P*(*D*), represents the likelihood of the data, and is calculated using standard methodology [62], and the numerator is calculated in a manner similar to that described previously (e.g., [7],[64],[65]). Equation 1 can further be used to compute the probability of a rate acceleration or a deceleration at a lineage simply by summing over *r*(*A*)*<r*(*B*) or *r*(*B*)*<r*(*A*), respectively. For each rate-shifting site, in order to determine whether a rate acceleration or deceleration occurred, we report the larger of the two probabilities. We note that since here we use an unrooted tree, a rate acceleration can also be interpreted as a rate deceleration, and vice-versa. Thus, in this study the terms rate acceleration and rate deceleration have meaning only in relation to one another.

### Simulations

Simulations were used in order to infer the false positive level of rate shift inference. To this end, we simulated 100 datasets under the assumption that no rate shift occurs by using the null model in this study (*P*(*ν* = 0)+*P*(*ν*→∞) = 1). In order to emulate realistic biological data, datasets' length and null-model parameters were based on the inference of the vpr protein. Each site was simulated along the tree of all subtypes used in this study (see section *Dataset*) using the JTT matrix [66]. For each dataset, the existence of rate shift was inferred with RASER using the LRT procedure described above, and rate-shifting positions were inferred computing posterior probabilities as described above.

### Structural Analysis

All available HIV-1 protein structures were obtained from the BioAfrica website (http://www.bioafrica.net). Thus, the following structures were used: Gag derived proteins: matrix p17, capsid p24, nucleocapsid p7 (PDB IDs: 1tam, 1e6j, 1a1t, respectively); Pol derived proteins: protease p10, reverse transcriptase p51, RNase p15, integrase p31 (PDB IDs: 1aaq, 1rtd, 1o1w, 2itg, respectively); Vpr p12/p10 (PDB ID: 1m8l); Tat p16/p14 (PDB ID: 1mnb); Rev p19 (PDB ID: 1etf); Vpu p16 (PDB ID: 1vpu); Env derived proteins: gp120, gp41 (PDB IDs: 1gc1, 1env, respectively); Nef p27/p25 (PDB ID: 2nef). For NMR determined structures the average over all models was used.

The accessible surface area (ASA) of each structure was computed using the Surface Racer program [67], with a probe radius of 1.4Å. Any residue was defined exposed to the solvent if its ASA exceeded 5% of its maximal (theoretical) ASA. The maximal ASA value of a residue was calculated in an extended GXG theoretical tripeptide, where G denotes glycine and X denotes the residue in question [68]. Secondary-structure assignments were obtained according to the dictionary of secondary structure of proteins [69]. G-test was used in order to determine whether rate-shifting sites are enriched with surface residues or certain secondary structure elements.

### Functional Analysis

Functional annotations of sites were retrieved from the Los Alamos sequence database (http://www.hiv.lanl.gov), as well as following a limited manual literature search.

### Dataset

Full genome sequences belonging to the nine subtypes (A, B, C, D, F, G, H, J, K) of HIV-1 group M were downloaded from the Los Alamos HIV sequence database (http://www.hiv.lanl.gov). Only sequences annotated as non-recombinants were selected, since recombinant sequences scramble the signal of the phylogeny (i.e., in recombinant sequences, different positions in the sequence have different phylogenies). Furthermore, the DSS method [70] for the detection of recombination was used to analyze the nine datasets, and no recombination was detected in any of the data. Sequences for which there was missing annotation for one of the nine ORFs of HIV-1 were removed. Furthermore, genomes for which one of the ORFs was annotated as either a pseudogene or a truncated sequence were removed. This yielded 64 A sequences, 147 B sequences, 224 C sequences, and 32 sequences from subtypes D, F, G, and J (no sequences of subtypes H and K were retained after the filtering process). Due to computational limitations, we sampled the 50 most distant sequences from subtypes A, B, and C. The genome of the reference sequence HXB2 was added on manually, and all sites described in this manuscript use this sequence as a reference. The genomes were separated into the 9 HIV-1 ORFs (see Table 1). Each ORF was aligned using the PRANK program version 080709 using the −F option [71]. At this stage, sequence AY901971 was removed due to poor alignment quality of the Vpu sequence. In total, this yielded 182 sequences. In order to reconstruct the phylogeny of these sequences, the alignments were concatenated. The reconstruction was performed with PhyML program version 2.4.5 [72] using among-site rate variation with 4 discrete rate categories, and the HIVb model [60] of sequence evolution, which was found to be the best-fit model for our dataset (see above). The phylogeny obtained showed that all seven subtypes were monophyletic, further validating that no recombinant viruses were erroneously obtained. In order to enhance the quality of the alignment, each ORF was next re-aligned with PRANK [71] using the phylogeny obtained as a guide tree.

### Implementation

RASER was implemented in C++. The program and source code are available at http://www.tau.ac.il/˜penn/raser.html.

## Supporting Information

Table S1 A list of all inferred rate-shifting sites, with information regarding secondary structure and exposed/buried classification.
(0.04 MB XLS)

Table S2 A list of all rate-shifting sites according to subtype, sorted according to acceleration/deceleration.
(0.06 MB XLS)

## References

[1] Wainberg MA (2004). HIV-1 subtype distribution and the problem of drug resistance.. AIDS.

[2] Simon F, Mauclere P, Roques P, Loussert-Ajaka I, Muller-Trutwin MC (1998). Identification of a new human immunodeficiency virus type 1 distinct from group M and group O.. Nat Med.

[3] Julg B, Goebel FD (2005). HIV genetic diversity: any implications for drug resistance?. Infection.

[4] Korber B, Gifford A, Myers G (1993). Patterns of variation among international isolates in the highly immunogenic V3 region of the HIV-1 envelope protein.. AIDS Res Hum Retroviruses.

[5] Gao F, Robertson DL, Carruthers CD, Morrison SG, Jian B (1998). A comprehensive panel of near-full-length clones and reference sequences for non-subtype B isolates of human immunodeficiency virus type 1.. J Virol.

[6] Travers SA, O'Connell MJ, McCormack GP, McInerney JO (2005). Evidence for heterogeneous selective pressures in the evolution of the env gene in different human immunodeficiency virus type 1 subtypes.. J Virol.

[7] Guindon S, Rodrigo AG, Dyer KA, Huelsenbeck JP (2004). Modeling the site-specific variation of selection patterns along lineages.. Proc Natl Acad Sci U S A.

[8] Kosakovsky Pond SL, Frost SDW, Grossman Z, Gravenor MB, Richman DD (2006). Adaptation to different human populations by HIV-1 revealed by codon-based analyses.. PLoS Comput Biol.

[9] Shafer RW, Eisen JA, Merigan TC, Katzenstein DA (1997). Sequence and drug susceptibility of subtype C reverse transcriptase from human immunodeficiency virus type 1 seroconverters in Zimbabwe.. J Virol.

[10] Fares MA, Travers SA (2006). A novel method for detecting intramolecular coevolution: adding a further dimension to selective constraints analyses.. Genetics.

[11] Ranga U, Shankarappa R, Siddappa NB, Ramakrishna L, Nagendran R (2004). Tat protein of human immunodeficiency virus type 1 subtype C strains is a defective chemokine.. J Virol.

[12] Bannister WP, Ruiz L, Loveday C, Vella S, Zilmer K (2006). HIV-1 subtypes and response to combination antiretroviral therapy in Europe.. Antivir Ther.

[13] Bocket L, Cheret A, Deuffic-Burban S, Choisy P, Gerard Y (2005). Impact of human immunodeficiency virus type 1 subtype on first-line antiretroviral therapy effectiveness.. Antivir Ther.

[14] Alexander CS, Montessori V, Wynhoven B, Dong W, Chan K (2002). Prevalence and response to antiretroviral therapy of non-B subtypes of HIV in antiretroviral-naive individuals in British Columbia.. Antivir Ther.

[15] Pillay D, Walker AS, Gibb DM, de Rossi A, Kaye S (2002). Impact of human immunodeficiency virus type 1 subtypes on virologic response and emergence of drug resistance among children in the Paediatric European Network for Treatment of AIDS (PENTA) 5 trial.. J Infect Dis.

[16] Kimura M (1983). Neutral Theory of Molecular Evolution.

[17] Knudsen B, Miyamoto MM (2001). A likelihood ratio test for evolutionary rate shifts and functional divergence among proteins.. Proc Natl Acad Sci U S A.

[18] Gu X (1999). Statistical methods for testing functional divergence after gene duplication.. Mol Biol Evol.

[19] Gaucher EA, Miyamoto MM, Benner SA (2001). Function–structure analysis of proteins using covarion-based evolutionary approaches: elongation factors.. Proc Natl Acad Sci U S A.

[20] Wang Y, Gu X (2001). Functional divergence in the caspase gene family and altered functional constraints: statistical analysis and prediction.. Genetics.

[21] Moreira D, Le Guyader H, Philippe H (1999). Unusually high evolutionary rate of the elongation factor 1 alpha genes from the Ciliophora and its impact on the phylogeny of eukaryotes.. Mol Biol Evol.

[22] Abhiman S, Sonnhammer EL (2005). Large-scale prediction of function shift in protein families with a focus on enzymatic function.. Proteins.

[23] Pupko T, Galtier N (2002). A covarion-based method for detecting molecular adaptation: application to the evolution of primate mitochondrial genomes.. Proc Biol Sci.

[24] Lopez P, Casane D, Philippe H (2002). Heterotachy, an important process of protein evolution.. Mol Biol Evol.

[25] Galtier N (2001). Maximum-likelihood phylogenetic analysis under a covarion-like model.. Mol Biol Evol.

[26] Yang Z, Nielsen R (2002). Codon-substitution models for detecting molecular adaptation at individual sites along specific lineages.. Mol Biol Evol.

[27] Zhang J, Nielsen R, Yang Z (2005). Evaluation of an improved branch-site likelihood method for detecting positive selection at the molecular level.. Mol Biol Evol.

[28] Gu X (2001). Maximum-likelihood approach for gene family evolution under functional divergence.. Mol Biol Evol.

[29] Gu X, Fu YX, Li WH (1995). Maximum likelihood estimation of the heterogeneity of substitution rate among nucleotide sites.. Mol Biol Evol.

[30] Blouin C, Boucher Y, Roger AJ (2003). Inferring functional constraints and divergence in protein families using 3D mapping of phylogenetic information.. Nucleic Acids Res.

[31] Dorman KS (2007). Identifying dramatic selection shifts in phylogenetic trees.. BMC Evol Biol.

[32] Nei M, Rogozin IB, Piontkivska H (2000). Purifying selection and birth-and-death evolution in the ubiquitin gene family.. Proc Natl Acad Sci U S A.

[33] Mayrose I, Doron-Faigenboim A, Bacharach E, Pupko T (2007). Towards realistic codon models: among site variability and dependency of synonymous and non-synonymous rates.. Bioinformatics.

[34] Berman HM, Westbrook J, Feng Z, Gilliland G, Bhat TN (2000). The Protein Data Bank.. Nucleic Acids Res.

[35] Lukashov VV, Huismans R, Rakhmanova AG, Lisitsina ZN, Akhtyrskaya NA (1999). Circulation of subtype A and gagA/envB recombinant HIV type 1 strains among injecting drug users in St. Petersburg, Russia, correlates with geographical origin of infections.. AIDS Res Hum Retroviruses.

[36] Carr JK, Zarandia M, Tsertsvadze T (2001). Distinctive subtype A HIV-1 in the former Soviet Union displays little diversity after six years of extensive geographic spread among IDU [abstract 197]..

[37] Novitsky VA, Montano MA, Essex M (1998). Molecular epidemiology of an HIV-1 subtype A subcluster among injection drug users in the Southern Ukraine.. AIDS Res Hum Retroviruses.

[38] Bobkov A, Cheingsong-Popov R, Selimova L, Ladnaya N, Kazennova E (1997). An HIV type 1 epidemic among injecting drug users in the former Soviet Union caused by a homogeneous subtype A strain.. AIDS Res Hum Retroviruses.

[39] Thomson MM, de Parga EV, Vinogradova A, Sierra M, Yakovlev A (2007). New insights into the origin of the HIV type 1 subtype A epidemic in former soviet union's countries derived from sequence analyses of preepidemically transmitted viruses.. AIDS Res Hum Retroviruses.

[40] Arvieux C, Tribut O (2005). Amprenavir or fosamprenavir plus ritonavir in HIV infection: pharmacology, efficacy and tolerability profile.. Drugs.

[41] Rusconi S, La Seta Catamancio S, Citterio P, Kurtagic S, Violin M (2000). Susceptibility to PNU-140690 (Tipranavir) of human immunodeficiency virus type 1 isolates derived from patients with multidrug resistance to other protease inhibitors.. Antimicrob Agents Chemother.

[42] Johnson VA, Brun-Vezinet F, Clotet B, Kuritzkes DR, Pillay D (2006). Update of the drug resistance mutations in HIV-1: Fall 2006.. Top HIV Med.

[43] Bohnlein E, Berger J, Hauber J (1991). Functional mapping of the human immunodeficiency virus type 1 Rev RNA binding domain: new insights into the domain structure of Rev and Rex.. J Virol.

[44] Pollard VW, Malim MH (1998). The HIV-1 Rev protein.. Annu Rev Microbiol.

[45] Novembre J, Galvani AP, Slatkin M (2005). The geographic spread of the CCR5 Δ32 HIV-resistance allele.. PLoS Biol.

[46] Rizzuto CD, Wyatt R, Hernandez-Ramos N, Sun Y, Kwong PD (1998). A conserved HIV gp120 glycoprotein structure involved in chemokine receptor binding.. Science.

[47] Robertson DL, Anderson JP, Bradac JA, Carr JK, Foley B (2000). HIV-1 nomenclature proposal.. Science.

[48] Middleton D, Williams F, Meenagh A, Daar AS, Gorodezky C (2000). Analysis of the distribution of HLA-A alleles in populations from five continents.. Hum Immunol.

[49] Williams F, Meenagh A, Darke C, Acosta A, Daar AS (2001). Analysis of the distribution of HLA-B alleles in populations from five continents.. Hum Immunol.

[50] Moore CB, John M, James IR, Christiansen FT, Witt CS (2002). Evidence of HIV-1 adaptation to HLA-restricted immune responses at a population level.. Science.

[51] Goulder PJ, Brander C, Tang Y, Tremblay C, Colbert RA (2001). Evolution and transmission of stable CTL escape mutations in HIV infection.. Nature.

[52] Leslie AJ, Pfafferott KJ, Chetty P, Draenert R, Addo MM (2004). HIV evolution: CTL escape mutation and reversion after transmission.. Nat Med.

[53] Bhattacharya T, Daniels M, Heckerman D, Foley B, Frahm N (2007). Founder effects in the assessment of HIV polymorphisms and HLA allele associations.. Science.

[54] Liu Y, McNevin J, Zhao H, Tebit DM, Troyer RM (2007). Evolution of human immunodeficiency virus type 1 cytotoxic T-lymphocyte epitopes: fitness-balanced escape.. J Virol.

[55] Fitch WM (1971). Toward defining the course of evolution: minimum change for a specific tree topology.. Syst Zool.

[56] Swofford DL, Olsen GJ, Waddell PJ, Hillis DM, Hillis DM, Mable BK (1996). Phylogenetic inference.. Molecular Systematics. 2nd ed.

[57] Yang Z (1996). Among-site variation and its impact on phylogenetic analyses.. Trends Ecol Evol.

[58] Yang Z (1994). Maximum likelihood phylogenetic estimation from DNA sequences with variable rates over sites: approximate methods.. J Mol Evol.

[59] Abascal F, Zardoya R, Posada D (2005). ProtTest: selection of best-fit models of protein evolution.. Bioinformatics.

[60] Nickle DC, Heath L, Jensen MA, Gilbert PB, Mullins JI (2007). HIV-specific probabilistic models of protein evolution.. PLoS ONE.

[61] Yang Z, Nielsen R, Goldman N, Pedersen AM (2000). Codon-substitution models for heterogeneous selection pressure at amino acid sites.. Genetics.

[62] Felsenstein J (1981). Evolutionary trees from DNA sequences: a maximum likelihood approach.. J Mol Evol.

[63] Self SG, Liang KY (1987). Asymptotic properties of maximum likelihood estimators and likelihood ratio tests under nonstandard conditions.. J Am Stat Assoc.

[64] Dutheil J, Pupko T, Jean-Marie A, Galtier N (2005). A model-based approach for detecting coevolving positions in a molecule.. Mol Biol Evol.

[65] Pupko T, Pe'er I, Shamir R, Graur D (2000). A fast algorithm for joint reconstruction of ancestral amino acid sequences.. Mol Biol Evol.

[66] Jones DT, Taylor WR, Thornton JM (1992). The rapid generation of mutation data matrices from protein sequences.. Comput Appl Biosci.

[67] Tsodikov OV, Record MT, Sergeev YV (2002). Novel computer program for fast exact calculation of accessible and molecular surface areas and average surface curvature.. J Comput Chem.

[68] Miller S, Janin J, Lesk AM, Chothia C (1987). Interior and surface of monomeric proteins.. J Mol Biol.

[69] Kabsch W, Sander C (1983). Dictionary of protein secondary structure: pattern recognition of hydrogen-bonded and geometrical features.. Biopolymers.

[70] McGuire G, Wright F, Prentice MJ (1997). A graphical method for detecting recombination in phylogenetic data sets.. Mol Biol Evol.

[71] Loytynoja A, Goldman N (2008). Phylogeny-aware gap placement prevents errors in sequence alignment and evolutionary analysis.. Science.

[72] Guindon S, Lethiec F, Duroux P, Gascuel O (2005). PHYML Online—a web server for fast maximum likelihood-based phylogenetic inference.. Nucleic Acids Res.

[73] Lum JJ, Cohen OJ, Nie Z, Weaver JG, Gomez TS (2003). Vpr R77Q is associated with long-term nonprogressive HIV infection and impaired induction of apoptosis.. J Clin Invest.

[74] Gatanaga H, Suzuki Y, Tsang H, Yoshimura K, Kavlick MF (2002). Amino acid substitutions in Gag protein at non-cleavage sites are indispensable for the development of a high multitude of HIV-1 resistance against protease inhibitors.. J Biol Chem.

[75] VerPlank L, Bouamr F, LaGrassa TJ, Agresta B, Kikonyogo A (2001). Tsg101, a homologue of ubiquitin-conjugating (E2) enzymes, binds the L domain in HIV type 1 Pr55(Gag).. Proc Natl Acad Sci U S A.

[76] Accola MA, Bukovsky AA, Jones MS, Gottlinger HG (1999). A conserved dileucine-containing motif in p6(gag) governs the particle association of Vpx and Vpr of simian immunodeficiency viruses SIV(mac) and SIV(agm).. J Virol.

[77] Svicher V, Ceccherini-Silberstein F, Erba F, Santoro M, Gori C (2005). Novel human immunodeficiency virus type 1 protease mutations potentially involved in resistance to protease inhibitors.. Antimicrob Agents Chemother.

[78] Wang WK, Dudek T, Essex M, Lee TH (1999). Hypervariable region 3 residues of HIV type 1 gp120 involved in CCR5 coreceptor utilization: therapeutic and prophylactic implications.. Proc Natl Acad Sci U S A.

[79] Kliger Y, Peisajovich SG, Blumenthal R, Shai Y (2000). Membrane-induced conformational change during the activation of HIV-1 gp41.. J Mol Biol.

